# Postbiotics and Dental Caries: A Systematic Review

**DOI:** 10.1002/cre2.70114

**Published:** 2025-03-11

**Authors:** Faezeh Heidari, Artak Heboyan, Dinesh Rokaya, Gustavo Vicentis Oliveira Fernandes, Mobina Heidari, Morteza Banakar, Muhammad Sohail Zafar

**Affiliations:** ^1^ Department of Pediatric Dentistry, Faculty of Dentistry Shahed University Tehran Iran; ^2^ Department of Research Analytics Saveetha Dental College and Hospitals, Saveetha Institute of Medical and Technical Sciences, Saveetha University Chennai India; ^3^ Department of Prosthodontics, Faculty of Stomatology Yerevan State Medical University after Mkhitar Heratsi Yerevan Armenia; ^4^ Department of Prosthodontics, School of Dentistry Tehran University of Medical Sciences Tehran Iran; ^5^ Clinical Sciences Department, College of Dentistry Ajman University Ajman United Arab Emirates; ^6^ Center of Medical and Bio‐Allied Health Sciences Research Ajman University Ajman United Arab Emirates; ^7^ A. T. Still University – Missouri School of Dentistry & Oral Health St. Louis Missouri USA; ^8^ Student Research Committee, School of Dentistry Shahid Sadoughi University of Medical Sciences Yazd Iran; ^9^ Health Policy Research Center, Institute of Health Shiraz University of Medical Sciences Shiraz Iran; ^10^ Dental Research Center, Dentistry Research Institute Tehran University of Medical Sciences Tehran Iran; ^11^ School of Dentistry, University of Jordan Amman Jordan

**Keywords:** cariogenic bacteria, dental caries, oral health, oral microbiome, postbiotics

## Abstract

**Objective:**

This systematic review aimed to evaluate the current evidence regarding the impact of postbiotics on dental caries, focusing on the effectiveness of postbiotic interventions in caries prevention, mechanisms of action, optimal dosages, and administration protocols.

**Methods:**

A literature search was conducted across PubMed/MEDLINE, Scopus, Web of Science, and the Cochrane Library. Eligible studies included randomized controlled trials, quasi‐experimental, observational, and in vitro studies. The selection followed the Preferred Reporting Items for Systematic Reviews and Meta‐Analyses guidelines. A qualitative synthesis was performed due to heterogeneity in study designs and outcomes.

**Results:**

Twenty‐one studies were included (18 in vitro and three randomized controlled trials). Postbiotics derived from various *Lactobacillus* species demonstrated inhibitory effects on *Streptococcus mutans* growth, biofilm formation, and virulence gene expression. Proposed mechanisms include direct antimicrobial activity, inhibition of bacterial adhesion, disruption of biofilm formation, modulation of immune responses, and pH buffering. After postbiotic interventions, human trials showed reduced salivary *S. mutans* counts and increased salivary pH.

**Conclusions:**

Postbiotics offer a promising novel approach to dental caries prevention by targeting cariogenic bacteria and modulating the oral microbiome through multiple mechanisms. Compared to probiotics, postbiotics present additional advantages, including enhanced safety, stability, and ease of incorporation into oral care products.

## 1 Introduction

Dental caries, a widespread chronic condition impacting individuals of all ages globally, arises from the intricate interactions of acidogenic bacteria, fermentable carbohydrates, and host factors throughout time (Kassebaum et al. 2017). Despite advancements in preventive measures and treatment strategies, the global burden of dental caries remains substantial, particularly in developing countries and among vulnerable populations (Peres et al. 2019). The complex etiology of dental caries involves various factors, including dietary habits, oral hygiene practices, and oral microbiome composition (Zhu et al. 2023).

Recently, the significance of the oral microbiota in the onset and advancement of dental caries has received heightened scrutiny (Moussa et al. 2022). The oral cavity harbors various microorganisms, including bacteria, fungi, and viruses, collectively forming the oral microbiome. The equilibrium between beneficial and pathogenic bacteria is essential for sustaining oral health, and dysbiosis of the oral microbiome has been associated with developing dental caries (Banakar et al. 2024; Sedghi et al. 2021). *Streptococcus mutans* is a key cariogenic bacterium, and following *S. mutans, Lactobacillus* can also be considered a critical bacteria that contributes to dental caries (Mallya and Mallya 2020). These bacteria produce acid, ultimately leading to the hydroxyapatite crystal's demineralization and caries (Zhang et al. 2022).

Traditional approaches to caries prevention and management have focused on the mechanical removal of dental plaque, fluoride application, and dietary modifications. However, these strategies have limitations, such as challenges with compliance, the rapid recolonization of bacteria after mechanical removal, the behavioral and socioeconomic barriers to dietary modifications, and their inability to address the underlying microbial imbalance driving caries development (Philip et al. 2018; Twetman 2018). So, there is a growing interest in exploring novel interventions that target the oral microbiome (Philip et al. 2018; Twetman 2018). Probiotics, live microorganisms that provide health benefits when properly provided, have demonstrated the potential to alter the oral microbiome and decrease the incidence of dental caries (Van Holm et al. 2023). However, using live microorganisms raises concerns regarding safety, stability, and potential side effects (Sanders et al. 2019). In contrast, postbiotics are nonviable bacterial metabolites or metabolic byproducts that have biological effects on the host (Aguilar‐Toalá et al. 2018). Postbiotics include cell‐free supernatants, cell lysates, secreted proteins, and metabolites produced by probiotic bacteria. They have many components, such as short‐chain fatty acids, enzymes, peptides, teichoic acids, cell surface proteins, and polysaccharides (Wegh et al. 2019). Postbiotics have gained attention as a potential alternative to probiotics, as they offer several advantages, such as improved safety profile, stability, longer shelf life, ease of incorporation into various delivery systems, and the ability to deliver targeted health benefits without the need for live microorganisms (Żółkiewicz et al. 2020).

The potential of postbiotics in promoting eubiosis, oral health, and preventing dental caries has been explored. For instance, postbiotic metabolites produced by *Lactobacillus* species have been shown to inhibit the growth and adherence of cariogenic bacteria and modulate the pH of the oral environment (Banakar et al. 2023; Elshaghabee et al. 2017; Jang et al. 2016). Additionally, postbiotics have been reported to modulate the immune response, reduce inflammation, and enhance the remineralization of tooth enamel (Cagetti et al. 2013; Luo et al. 2024). However, despite the growing interest in postbiotics and their potential applications in oral health, there is a lack of comprehensive and systematic evidence synthesizing their impact on dental caries. The heterogeneity in study designs, population characteristics, and outcome measures across various studies complicates the ability to determine the effectiveness of postbiotics in preventing and managing dental caries (Banakar et al. 2024).

Considering the constraints of prior studies and the increasing interest in postbiotics as an innovative strategy for caries prevention and management, there is a distinct necessity for a systematic review that particularly investigates the effects of postbiotics on dental caries. This systematic review seeks to rigorously assess the existing evidence about the effects of postbiotics on dental caries. The review evaluates the efficacy of diverse postbiotic therapies in preventing dental caries, their possible mechanisms of action, and the ideal dosage, frequency, and duration of postbiotic administration. This study synthesizes available data, offering significant insights for researchers, dental practitioners, and policymakers, thereby aiding the development of creative solutions for preventing and managing dental caries.

## 2 Methods

### 2.1 Search Strategy

A comprehensive literature search was conducted in multiple electronic databases, including PubMed, Scopus, Web of Science, and Cochrane Library, from inception to October 2024. The search strategy was developed in collaboration with a medical librarian to ensure the inclusion of all relevant studies. A combination of Medical Subject Headings (MeSH) terms and free‐text keywords were used, including “postbiotics,” “parabiotics,” “cell‐free supernatants,” “bacterial lysates,” “bacterial metabolites,” “dental caries,” “tooth decay,” “cariogenic bacteria,” “Streptococcus mutans,” “lactobacilli,” “caries prevention,” “oral health,” and their synonyms. These terms will be combined using Boolean operators and adjusted for each database to ensure maximum retrieval efficiency (Salvador‐Oliván et al. 2019). The search was limited to studies published in English. Additionally, the reference lists of included studies and relevant systematic reviews were manually screened to identify any additional eligible studies.

### 2.2 Eligibility Criteria

The study selection process followed the Preferred Reporting Items for Systematic Reviews and Meta‐Analyses (PRISMA) 2020 guidelines (Page et al. 2021). Table 1 summarizes the eligibility criteria for this study. The inclusion criteria for this systematic review were as follows: (1) randomized controlled trials (RCTs), quasi‐experimental studies, observational studies, or in vitro studies; (2) studies investigating the impact of postbiotics on dental caries or cariogenic bacteria; (3) studies reporting at least one outcome measure related to dental caries, such as caries incidence, cariogenic bacteria count or related genes expression.

**Table 1:** Inclusion and exclusion criteria.

Criteria	Inclusion criteria	Exclusion criteria
Study design	RCTs, quasi‐experimental, observational, in vitro	Reviews, editorials, abstracts
Population	Studies on dental caries or cariogenic bacteria	Studies on conditions unrelated to dental caries
Intervention	Postbiotics or their effects on caries/bacteria	Probiotics or interventions not classified as postbiotics
Outcome measures	Caries incidence, bacterial count, gene expression	Studies without relevant outcomes or focus on oral conditions other than dental caries

Studies were excluded if they (1) were non‐original research articles, such as reviews, editorials, or conference abstracts; (2) involved the use of probiotics or other interventions not classified as postbiotics; (3) focused on oral conditions other than dental caries.

### 2.3 Study Selection

The study selection process involved two stages. First, two independent reviewers screened the titles and abstracts of all records identified through the search strategy. Second, the full‐text articles of potentially eligible studies were retrieved and assessed against the inclusion criteria. Any disagreements with the reviewer were resolved through discussion or consulting a third reviewer if necessary. The reasons for excluding studies during the full‐text screening stage were documented.

### 2.4 Data Collection and Data Items

Two independent reviewers extracted data from the included studies using a standardized data extraction form. The extracted data included study characteristics (e.g., first author, publication year, study design, sample size, and follow‐up duration), participant characteristics (e.g., age, gender, and baseline caries status), intervention details (e.g., type of postbiotic, dosage, frequency, and duration of administration), comparison group details, outcome measures (e.g., caries incidence, cariogenic bacteria count, or related gene expression), and main findings. Any discrepancies in the extracted data were resolved through discussion or consulting a third reviewer if necessary. The study authors were contacted for clarification or additional information if the required data were unclear or missing.

### 2.5 Data Synthesis

A qualitative synthesis of the included studies was performed due to the heterogeneity in study design, participants, interventions, and outcome measures, precluding the feasibility of conducting a meta‐analysis. The studies were grouped according to the type of postbiotic investigated, and the findings were summarized narratively, focusing on the impact of postbiotics on dental caries and cariogenic bacteria. The narrative synthesis included a description of the study characteristics, participant characteristics, intervention details, and main findings related to the outcomes of interest. The limitations and potential sources of bias in the included studies were also discussed.

## 3 Results

### 3.1 Study Characteristics

**Figure 1:**
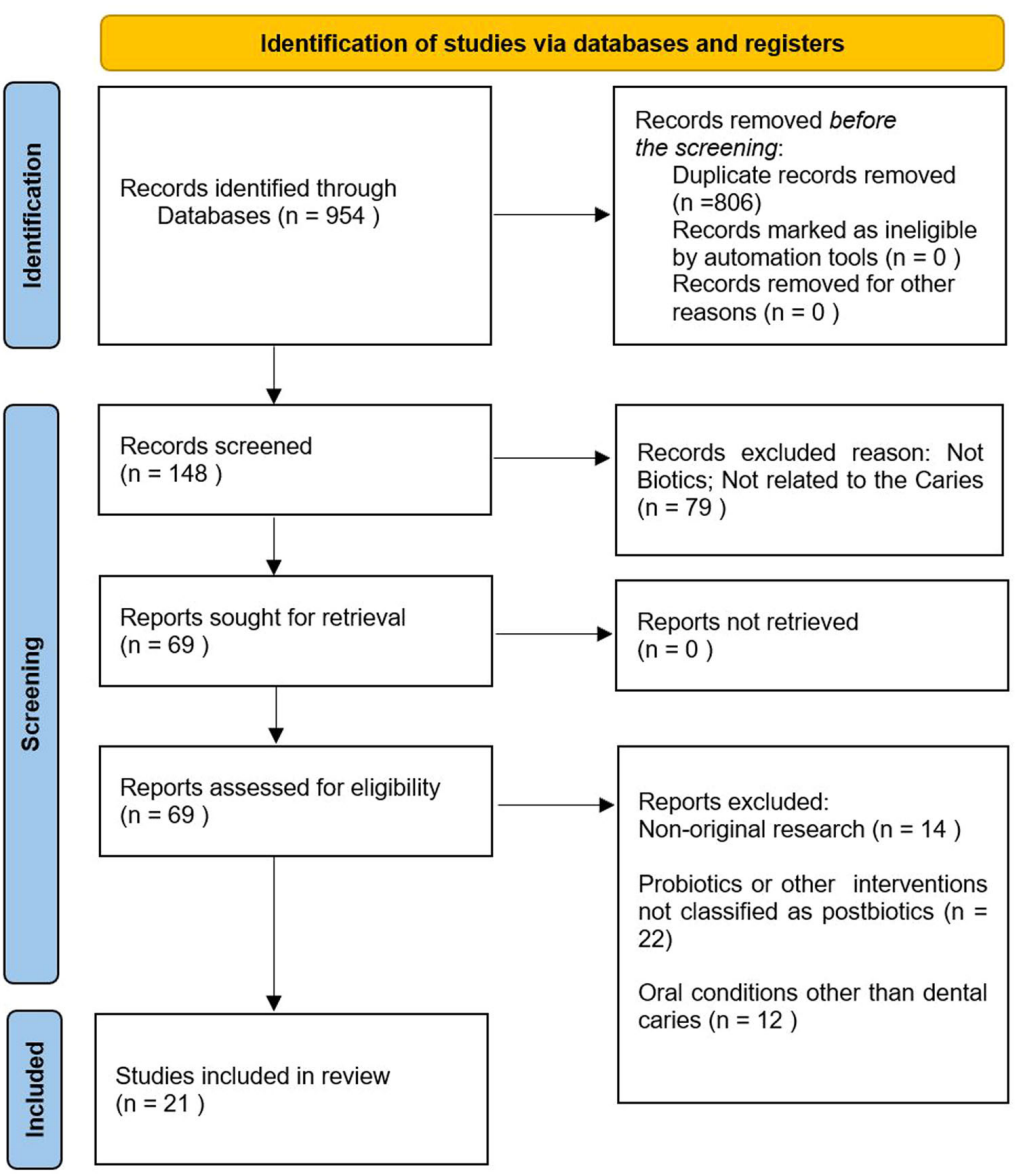
PRISMA flowchart for screened records.

A PRISMA flowchart indicated the number of studies identified, screened, evaluated for eligibility, and included in the final evaluation (Figure 1). The systematic search produced 954 potentially related articles. After eliminating duplicates and evaluating titles and abstracts, 148 full‐text articles were examined for eligibility. Twenty‐one studies fulfilled the inclusion criteria and were incorporated into the systematic review.

This systematic review encompassed 21 papers examining the effects of postbiotics on dental caries (Table 2). The studies were published between 2014 and 2023, reflecting the growing interest in this research area in recent years (Ahn et al. 2018; Banakar et al. 2023; Basir et al. 2022; Chen et al. 2020; Ciandrini et al. 2016; Jeong et al. 2018; Jung et al. 2021; Kim, Jang, et al. 2022; Kim, Hyun, et al. 2022; Lin et al. 2022; Liu et al. 2024; OmerOglou et al. 2022; Park et al. 2023; Rossoni et al. 2018; Salehi et al. 2014; Savabi et al. 2014; Srivastava et al. 2020; Tahmourespour et al. 2019; Wasfi et al. 2018; Yang et al. 2021; Zhao et al. 2023).

**Table 2:** Details from the included articles.

Author (Year)	Study design	Postbiotic source	Postbiotic type	Intervention details	Main findings	Limitations
Savabi et al. (2014)	In vitro	*Lactobacillus casei*	Biosurfactant	Exposure of *S. mutans* to varying concentrations of biosurfactant for 24 h	Biosurfactant significantly inhibited *S. mutans* growth and reduced the expression of *gtfB*, *gtfC*, and *ftf* genes involved in biofilm formation.	In vitro study; limited generalizability to clinical settings.
Salehi et al. (2014)	In vitro	*L. reuteri*	Biosurfactant	Exposure of *S. mutans* to varying concentrations of biosurfactant for 24 h	Biosurfactant significantly inhibited *S. mutans* growth and reduced the expression of *gtfB*, *gtfC*, and *ftf* genes involved in biofilm formation.	In vitro study; limited generalizability to clinical settings.
Ciandrini et al. (2016)	In vitro	*L. reuteri* *L. acidophilus* *L. rhamnosus* *L. paracasei*	Biosurfactants	Exposure of oral streptococci biofilms to biosurfactants	Biosurfactants from different Lactobacillus strains exhibited varying inhibitory activity against oral streptococci's growth and biofilm formation.	In vitro study; limited generalizability to clinical settings.
Ahn et al. (2018)	In vitro	*L. plantarum*	Lipoteichoic acid	Exposure of *S. mutans* to LTA for 24 h	LTA inhibited *S. mutans* growth, biofilm formation, and adherence to saliva‐coated hydroxyapatite in a dose‐dependent manner.	In vitro study; limited generalizability to clinical settings.
Jeong et al. (2018)	In vitro	*L. kefiranofaciens* *L. plantarum* *L. johnsonii* *L. rhamnosus*	Cell‐free supernatant	Exposure of oral pathogens, including *S. mutans and S. sobrinus*, to CFS	CFS effectively inhibited the growth and biofilm formation of *S. mutans* and *S. sobrinus*. Inhibited the expression of *ftf*, *gtfB*, *gtfC* (by *Lactobacillus kefiranofaciens*)	In vitro study; limited generalizability to clinical settings.
Rossoni et al. (2018)	In vitro	Various *Lactobacillus* spp. *(L. paracasei, L. fermentum and L. rhamnosus)*	Cell‐free supernatant	Exposure of *S. mutans* biofilms to CFS for 48 h	Inhibition of *S. mutans* biofilm formation and reduction in preformed biofilms by CFS from various oral *Lactobacillus* strains.	In vitro study; limited generalizability to clinical settings.
Wasfi et al. (2018)	In vitro	*L. casei* *L. reuteri* *L. plantarum* *L. salivarius*	Cell‐free supernatant	Exposure of *S. mutans* to CFS	CFS from selected *Lactobacillus* strains inhibited *S. mutans* growth, biofilm formation, and the expression of genes related to adhesion, acidogenicity, and exopolysaccharide production.	In vitro study; limited generalizability to clinical settings.
Tahmourespour et al. (2019)	In vitro	*L. rhamnosus*	Biosurfactant	Exposure of *S. mutans* to biosurfactant	Inhibition of *S. mutans* biofilm formation and downregulation of *gtfB*, *gtfC*, and *ftf* genes.	In vitro study; limited generalizability to clinical settings.
Chen et al. (2020)	In vitro	*L. reuteri*	Cell‐free supernatant	Exposure of multispecies oral biofilms to CFS	CFS altered the composition and architecture of the cariogenic biofilm, reducing the abundance of *S. mutans* and other cariogenic species. It also reduced enamel demineralization, indicating a protective effect against caries.	In vitro study; limited generalizability to clinical settings.
Srivastava et al. (2020)	In vitro	*L. plantarum*	Cell‐free supernatant	Exposure of *S. mutans* and *Candida albicans* biofilms to CFS	Inhibition of mixed‐species biofilm formation and reduction in preformed biofilms. Downregulation of *GtfB*, *gtfC*, and *gtfD* gene expression in *S. mutans* biofilms Downregulation of *HWP1*, *ALS1*, and *ALS3* gene expression in *C. albicans* biofilms	In vitro study; limited generalizability to clinical settings.
Jung et al. (2021)	In vitro	*L. plantarum* *L. paracasei* *L. casei* *L. rhamnosus* *L. salivarius* *L. lactis* *L. fermentum*	Cell‐free supernatant	Exposure of *S. mutans* biofilms to CFS	Inhibition of biofilm formation and reduction in the expression of genes involved in biofilm formation and acidogenicity.	In vitro study; limited generalizability to clinical settings.
Yang et al. (2021)	In vivo	*L. reuteri*	Cell‐free supernatant	Exposure of *Porphyromonas gingivalis*, *Fusobacterium nucleatum*, and *Streptococcus mutans* to CFS	CSF significantly reduced growth rate, intracellular ATP levels, cell viability, and time‐to‐kill of the oral pathogens. CSF also disrupted biofilm formation and suppressed gene expression related to biofilm formation in *P. gingivalis*. The antibacterial activity of CSF is attributed to fatty acids and/or sugars.	In vitro study; limited generalizability to clinical settings.
Kim, Jang, et al. (2022)	In vitro	*L. brevis*	Cell‐free supernatant	Exposure of *S. mutans* to CFS	CFS significantly inhibited the growth and biofilm formation of *S. mutans* in a dose‐dependent manner.	In vitro study; limited generalizability to clinical settings.
Kim, Hyun, et al. (2022)	In vitro	*L. plantarum* *L. rhamnosus GG*	Bacterial lysates	Exposure of oral *Streptococcus mutans*, *Porphyromonas gingivalis*, *Fusobacterium nucleatum* to bacterial lysates	Bacterial lysates from selected *Lactobacillus* strains exhibited anti‐inflammatory and antibiofilm activities against oral pathogens.	In vitro study; limited generalizability to clinical settings.
OmerOglou et al. (2022)	In vitro	*L. curvatus*, *L. acidophilus*	Cell‐free supernatant	Exposure of *S. mutans* to CFS	Reduced biofilm formation and expression of virulence genes involved in quorum sensing and biofilm formation.	In vitro study; limited generalizability to clinical settings.
Banakar et al. (2023)	In vitro	*L. rhamnosus* GG, *L. reuteri*	Cell‐free supernatant	Exposure of *S. mutans* to CFS	CFS from both strains exhibited significant antimicrobial activity against *S. mutans*, inhibiting its growth and biofilm formation. Additionally, CFS downregulated the expression of *gtfB*, *gtfC*, and *ftf* genes involved in *S. mutans* virulence.	In vitro study; limited generalizability to clinical settings.
Basir et al. (2022)	Randomized controlled trial	*L. paracasei*	Toothpaste containing postbiotics	Daily use of toothpaste for 4 weeks in children aged 6–12 years	Postbiotic toothpaste significantly increased salivary IgA levels and pH compared to fluoride toothpaste. It also reduced salivary *S. mutans* counts, although the difference was insignificant.	Small sample size; limited generalizability to other populations.
Lin et al. (2022)	Randomized controlled trial	*L. paracasei*	Heat‐killed probiotic and postbiotic oral lozenges	Daily consumption of lozenges for 4 weeks in adults with dental caries	The intervention group showed a significant reduction in salivary *S. mutans* counts and plaque index compared to the placebo group.	Small sample size; limited generalizability to other populations.
Liu et al. (2024)	Randomized controlled trial	Probio‐Eco: *Lacticaseibacillus casei Zhang* *Lactiplantibacillus plantarum* *Bifidobacterium anlmalis subsp. lactis*	Tablets (Inactivated bacteria)	Tablets twice daily for 14 days (one tablet after lunch and dinner)	Postbiotic intervention after dental filling improved caries prognosis by restoring oral microbiota diversity, structure, and function and reducing potentially pathogenic bacteria.	Reliance on prediction algorithms (BugBase and PICRUSt2) for functional analysis, requiring further validation. Relatively short intervention duration (14 days).
Park et al. (2023)	In vitro	*Limosilactobacillus fermentum*	Cell‐free supernatant	Exposure of *S. mutans* to CFS	CFS from selected *L. fermentum* strains effectively inhibited the growth and biofilm formation of *S. mutans*.	In vitro study; limited generalizability to clinical settings.
Zhao et al. (2023)	In vitro	*L. paracasei*	Cell‐free supernatant	Exposure of *S. mutans* to CFS	Inhibited the formation of *S. mutans* biofilms and reduced the expression of genes involved in biofilm formation.	In vitro study; limited generalizability to clinical settings.

Abbreviations: CFS, cell‐free supernatants; LTA, lipoteichoic acid.

The majority of the included studies (*n* = 17) employed experimental designs to evaluate the effects of postbiotics on cariogenic bacteria and biofilm formation (Ahn et al. 2018; Banakar et al. 2023; Chen et al. 2020; Ciandrini et al. 2016; Jeong et al. 2018; Jung et al. 2021; Kim et al. 2022; OmerOglou et al. 2022; Park et al. 2023; Rossoni et al. 2018; Salehi et al. 2014; Savabi et al. 2014; Srivastava et al. 2020; Tahmourespour et al. 2019; Wasfi et al. 2018; Yang et al. 2021; Zhao et al. 2023). The in vitro studies primarily used reference strains of *S. mutans*, the main cariogenic bacterium. Three randomized controlled trials were conducted in human populations, including children aged 6–12 years (Basir et al. 2022) and adults with dental caries (Lin et al. 2022; Liu et al. 2024).

### 3.2 Postbiotic Interventions

#### 3.2.1 Types of Postbiotics Used

The postbiotics investigated in the included studies were derived from various *Lactobacillus* species, such as *L. rhamnosus*, *L. plantarum*, *L. reuteri*, *L. fermentum*, *L. acidophilus*, *L. paracasei*, *L. salivarius*, *L. kefiranofaciens*, and *L. casei*. The postbiotics were administered in different forms: cell‐free supernatants, purified lipoteichoic acid, biosurfactants, bacterial lysates, and toothpaste containing postbiotics.

#### 3.2.2 Dosage and Duration of Interventions

The dosages and duration of postbiotic therapies differed among the research examined. Numerous in vitro investigations evaluated various concentrations to ascertain the minimum inhibitory concentration (MIC) and minimum bactericidal concentration (MBC) of postbiotics against *S. mutans*. The exposure length ranged from 24 h to 7 days in these studies (Ahn et al. 2018; Banakar et al. 2023; Chen et al. 2020; Ciandrini et al. 2016; Jeong et al. 2018; Jung et al. 2021; Kim et al. 2022; OmerOglou et al. 2022; Park et al. 2023; Rossoni et al. 2018; Salehi et al. 2014; Savabi et al. 2014; Srivastava et al. 2020; Tahmourespour et al. 2019; Wasfi et al. 2018; Yang et al. 2021; Zhao et al. 2023). The human trials provided postbiotics daily for 2‐4 weeks, either as toothpaste (Basir et al. 2022) or tablets (Lin et al. 2022; Liu et al. 2024).

### 3.3 Effects on Dental Caries

Most of the included studies demonstrated that postbiotics derived from *Lactobacillus* species effectively reduced the incidence and severity of dental caries in vitro. In vitro studies showed that postbiotics significantly inhibited the growth and biofilm formation of *S. mutans* (Ahn et al. 2018; Banakar et al. 2023; Jung et al. 2021; Kim et al. 2022; Liu et al. 2024; OmerOglou et al. 2022; Park et al. 2023; Rossoni et al. 2018; Srivastava et al. 2020; Wasfi et al. 2018; Yang et al. 2021; Zhao et al. 2023). Postbiotics also reduced the expression of virulence genes involved in *S. mutans* adhesion, acid production, and exopolysaccharide synthesis (Banakar et al. 2023; Liu et al. 2024; OmerOglou et al. 2022; Salehi et al. 2014; Savabi et al. 2014; Tahmourespour et al. 2019; Zhao et al. 2023). The human trials found that postbiotic interventions reduced salivary *S. mutans* counts (Basir et al. 2022; Lin et al. 2022; Liu et al. 2024) and increased salivary pH (Basir et al. 2022), indicating a lower caries risk (Basir et al. 2022; Lin et al. 2022; Liu et al. 2024).

Several potential mechanisms have been proposed to explain the anticaries effects of postbiotics:

1. **Direct antimicrobial activity:** Some compounds are present in postbiotics, namely, organic acids, hydrogen peroxide, and bacteriocins; those can inhibit the growth of cariogenic bacteria like *S. mutans* (Kim et al. 2022; Kim et al. 2022; Park et al. 2023; Rossoni et al. 2018; Wasfi et al. 2018).
2. **Inhibition of bacterial adhesion:** The mechanism in which postbiotics interfere with *S. mutans* adhesion to the tooth surface may be at a competitive level either by competing for the binding sites or altering surface properties of the bacteria (Ahn et al. 2018; Ciandrini et al. 2016; Salehi et al. 2014; Savabi et al. 2014; Tahmourespour et al. 2019).
3. **Disruption of biofilm formation:** Postbiotics can inhibit the synthesis of exopolysaccharides, one of the essential parts in the matrix composition of *S. mutans* biofilms, through downregulating genes of glucosyltransferases encoding enzymes such as *gtfB*, *gtfC*, and a gene fructosyltransferase encoding enzyme – *ftf* (Banakar et al. 2023; Jeong et al. 2018; Liu et al. 2024; OmerOglou et al. 2022; Salehi et al. 2014; Savabi et al. 2014; Tahmourespour et al. 2019; Zhao et al. 2023).
4. **Modulation of immune responses:** Some postbiotics have been reported to enhance the production of salivary immunoglobulin A (IgA), which can neutralize *S. mutans* and inhibit its adhesion to tooth surfaces (Basir et al. 2022; Lin et al. 2022).
5. **Buffering of acidic pH:** The production of postbiotics may keep the oral pH neutral either by the production of alkaline compounds or buffering of acids produced by cariogenic bacteria, thereby preventing enamel demineralization (Basir et al. 2022; Liu et al. 2024).

## 4 Discussion

This systematic review synthesized the current evidence on the impact of postbiotics on dental caries prevention. The majority of the included studies demonstrated that postbiotics effectively reduced the growth and biofilm formation of *S. mutans* in vitro (Ahn et al. 2018; Banakar et al. 2023; Jung et al. 2021; Kim et al. 2022; Liu et al. 2024; OmerOglou et al. 2022; Park et al. 2023; Rossoni et al. 2018; Srivastava et al. 2020; Wasfi et al. 2018; Yang et al. 2021; Zhao et al. 2023). The mechanisms underlying the anticaries effects of postbiotics are multifaceted and involve direct antimicrobial activity, inhibition of bacterial adhesion, disruption of biofilm formation, modulation of immune responses, and buffering of acidic pH (Ahn et al. 2018; Basir et al. 2022; Ciandrini et al. 2016; Kim et al. 2022; Kim et al. 2022; Lin et al. 2022; Liu et al. 2024; Park et al. 2023; Rossoni et al. 2018; Salehi et al. 2014; Savabi et al. 2014; Tahmourespour et al. 2019; Wasfi et al. 2018).

The inhibitory effects of postbiotics on *S. mutans* biofilm formation may be attributed to their ability to interfere with bacterial adhesion mechanisms and disrupt the synthesis of exopolysaccharides, which are essential components of the biofilm matrix (Banakar et al. 2023; Liu et al. 2024; OmerOglou et al. 2022; Salehi et al. 2014; Savabi et al. 2014; Tahmourespour et al. 2019; Zhao et al. 2023). Moreover, postbiotics have been shown to modulate the expression of virulence genes involved in *S. mutans* adhesion, acid production, and exopolysaccharide synthesis (Banakar et al. 2023; Liu et al. 2024; OmerOglou et al. 2022; Salehi et al. 2014; Savabi et al. 2014; Tahmourespour et al. 2019; Zhao et al. 2023). This modulation of gene expression may lead to a less virulent bacterial phenotype, potentially reducing the cariogenic potential of *S. mutans* in the oral cavity. The antimicrobial activity of postbiotics against *S. mutans* and other oral pathogens is another important aspect of their anticaries effects. Several studies demonstrated that postbiotics produced by *Lactobacillus* species contain antimicrobial compounds such as organic acids, hydrogen peroxide, and bacteriocins (Kim et al. 2022; Park et al. 2023; Rossoni et al. 2018; Wasfi et al. 2018). It was proposed that these chemicals can directly limit the growth of cariogenic bacteria, hence diminishing their populations in the oral microbiome. An additional intriguing prospect is the potential regulation of the immune response in the oral cavity using postbiotics. Several studies indicate that postbiotics may enhance the synthesis of salivary IgA, which is crucial for neutralizing oral infections and inhibiting their attachment to dental surfaces (Basir et al. 2022; Lin et al. 2022). This immunomodulatory effect of postbiotics may contribute to their overall anticaries activity by enhancing the host's natural defense mechanisms against cariogenic bacteria. The ability of postbiotics to maintain a neutral pH in the oral cavity is another important factor in their anticaries effects. Some studies have shown that postbiotics can help buffer acids produced by cariogenic bacteria, thereby preventing enamel demineralization (Basir et al. 2022; Liu et al. 2024). This pH‐modulating effect of postbiotics may be particularly beneficial in individuals with high caries risk or those with reduced salivary flow.

**Figure 2:**
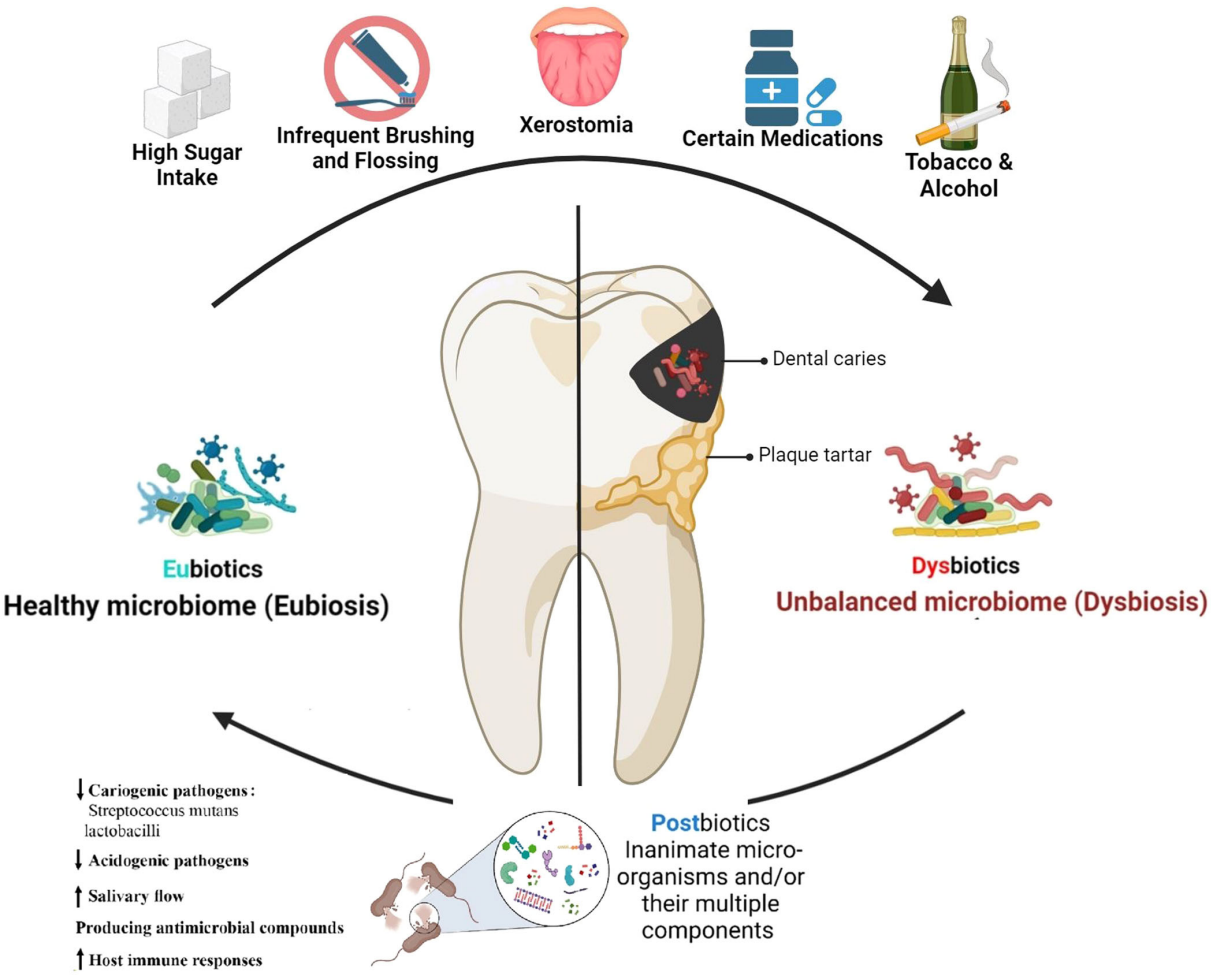
A diagram illustrating a sequence in which lifestyle‐related factors may contribute to the transition into microbiome dysbiosis, subsequently leading to numerous dental issues: sugar consumption, inadequate oral hygiene, xerostomia, pharmacological interventions, and the use of tobacco or alcohol. The illustration depicts the transition of microbiota from a healthy condition to an imbalanced state; postbiotics, encompassing nonliving bacteria and their metabolic byproducts, play a role in sustaining dental health.

The findings indicate that postbiotics might represent a new strategy for preventing and treating dental caries by promoting eubiosis and perturbing key pathogenic mechanisms in caries development (Figure 2). These findings add to the growing literature on the oral microbiome's role in dental caries and new microbiome‐targeted therapies. Previous reviews have highlighted the promise of probiotics in caries prevention but also noted challenges associated with using live microorganisms, such as safety concerns and viability issues (Banakar et al. 2024; Cagetti et al. 2013; Twetman 2018). One of the advantages of postbiotics over probiotics is their improved safety profile and stability. In contrast to live probiotic bacteria, postbiotics are nonviable bacterial products or metabolites, hence alleviating worries regarding the potential colonization of probiotic strains in the oral cavity or systemic infections in immunocompromised persons (Aguilar‐Toalá et al. 2018; Żółkiewicz et al. 2020). Also, postbiotics may have a longer shelf life and be easier to incorporate into oral care products, such as toothpaste, mouthwashes, or lozenges (Banakar et al. 2024).

Postbiotics, a promising treatment for oral health, face several challenges in clinical implementation. These include the lack of standardized formulations and dosing protocols, underdeveloped regulatory frameworks, scalability of postbiotic production, cost‐effectiveness compared to conventional therapies, and long‐term safety data in diverse populations. Ensuring long‐term stability in oral care products like toothpaste and mouthwash is a significant formulation challenge. The limited availability of large‐scale, long‐term human clinical trials restricts widespread adoption, as robust evidence is needed to demonstrate efficacy and safety across different demographic groups. The cost‐effectiveness of postbiotic‐based interventions compared to traditional fluoride therapies must be evaluated in future research to justify their integration into routine dental care. Collaborative efforts between researchers, industry stakeholders, and regulatory bodies are crucial to overcoming these challenges and integrating postbiotics into mainstream oral healthcare (Aguilar‐Toalá et al. 2018; Żółkiewicz et al. 2020; Banakar et al. 2024).

One of the major strengths of this systematic review is the comprehensive search strategy involving multiple electronic databases and a thorough manual screening of reference lists to identify all relevant studies. Additionally, including both in vitro and in vivo studies provided a more comprehensive understanding of the anticaries effects of postbiotics and their potential mechanisms of action. While the results of this systematic review are promising, several limitations should be considered. First, most of the included studies were in vitro experiments, which may not fully reflect the complex interactions in the oral cavity. Second, the human trials included in this review had relatively short follow‐up periods, making it difficult to assess the long‐term effects of postbiotics on caries prevention. Third, there was considerable heterogeneity in the types of postbiotics used, postbiotic preparations, forms of administration (e.g., toothpaste, lozenges, cell‐free supernatants), their dosages, and the duration of interventions across studies, making it challenging to draw definitive conclusions about the optimal postbiotic regimens for caries prevention. Additionally, there is a lack of standardized outcome measures, as different studies used various metrics such as *S. mutans* counts, biofilm formation assays, gene expression analyses, and clinical indices, thereby complicating direct comparisons and synthesis of results. These heterogeneity hamper the ability to perform meta‐analyses and to develop standardized recommendations.

Future research directions should include large‐scale, long‐term, randomized controlled trials to evaluate the efficacy of postbiotics in preventing and managing dental caries in different population groups, including children, adults, and individuals at high caries risk. Additionally, studies should investigate the effects of postbiotics on the overall oral microbiome composition and diversity rather than focusing solely on *S. mutans*. There is a continued necessity to explore the potential synergistic effects of integrating postbiotics with other preventive measures, such as fluoride or xylitol. Mechanistic studies should be conducted, particularly concerning alterations in the host immune response induced by postbiotics and their role in caries prevention within the oral cavity. Ultimately, research should be undertaken to develop novel postbiotic delivery strategies that enhance retention and improve efficacy within the oral cavity.

## 5 Conclusion

Postbiotics have shown potential in preventing and managing dental caries through a multisectoral mechanism that inhibits the growth of cariogenic bacteria, modulates oral microbiota, enhances host immunity, and maintains neutral mouth pH. They offer advantages over traditional probiotics, such as increased safety for sensitive consumers and patients, better stability during storage, and broader opportunities for incorporation into oral care products. Postbiotics act against pathogenic mechanisms at the root of caries development, potentially enhancing existing preventive approaches. However, further clinical testing is needed, and future research should focus on large‐scale randomized controlled trials to provide standardized optimal postbiotic formulation, dosage, and administration guidelines. Long‐term efficacy and safety assessments in diverse populations, considering age, dietary habits, and baseline oral health status, could position postbiotics as a crucial part of comprehensive caries prevention strategies, reducing disease prevalence and improving oral health worldwide.

## Author Contributions

*Conceptualization*: Faezeh Heidari, Mobina Heidari, Morteza Banakar, and Muhammad Sohail Zafar. *Methodology*: Faezeh Heidari, Artak Heboyan, Dinesh Rokaya, Gustavo Vicentis Oliveira Fernandes, Mobina Heidari, Morteza Banakar, and Muhammad Sohail Zafar. *Software*: Faezeh Heidari, Artak Heboyan, Morteza Banakar, and Muhammad Sohail Zafar. *Validation*: Faezeh Heidari, Artak Heboyan, Dinesh Rokaya, Gustavo Vicentis Oliveira Fernandes, Mobina Heidari, Morteza Banakar, and Muhammad Sohail Zafar. *Formal analysis*: Faezeh Heidari, Artak Heboyan, Dinesh Rokaya, Gustavo Vicentis Oliveira Fernandes, Mobina Heidari, Morteza Banakar, and Muhammad Sohail Zafar. *Investigation*: Faezeh Heidari, Artak Heboyan, Dinesh Rokaya, Gustavo Vicentis Oliveira Fernandes, Mobina Heidari, Morteza Banakar, and Muhammad Sohail Zafar. *Resources*: Faezeh Heidari, Artak Heboyan, Dinesh Rokaya, Gustavo Vicentis Oliveira Fernandes, Mobina Heidari, Morteza Banakar, and Muhammad Sohail Zafar. *Data curation*: Faezeh Heidari, Artak Heboyan, Dinesh Rokaya, Gustavo Vicentis Oliveira Fernandes, Mobina Heidari, Morteza Banakar, and Muhammad Sohail Zafar. *Writing – original draft preparation*: Faezeh Heidari, Artak Heboyan, Dinesh Rokaya, Gustavo Vicentis Oliveira Fernandes, Mobina Heidari, Morteza Banakar, and Muhammad Sohail Zafar. *Writing – review and editing*: Faezeh Heidari, Artak Heboyan, Dinesh Rokaya, Gustavo Vicentis Oliveira Fernandes, Mobina Heidari, Morteza Banakar, and Muhammad Sohail Zafar. *Visualization*: Faezeh Heidari, Artak Heboyan, Dinesh Rokaya, Gustavo Vicentis Oliveira Fernandes, Mobina Heidari, Morteza Banakar, and Muhammad Sohail Zafar. *Supervision*: Morteza Banakar and Muhammad Sohail Zafar. *Project administration*: Faezeh Heidari, Morteza Banakar, and Muhammad Sohail Zafar. All authors have read and agreed to the published version of the manuscript.

## Conflicts of Interest

The authors declare no conflicts of interest.

## Data Availability

The data sets used and/or analyzed during the current study are available from the corresponding author upon reasonable request.
